# Effect of mechanical ventilation during cardiopulmonary bypass on end-expiratory lung volume in the perioperative period of cardiac surgery: an observational study

**DOI:** 10.1186/s13019-022-02063-7

**Published:** 2022-12-22

**Authors:** Léa Trancart, Nathalie Rey, Vincent Scherrer, Véronique Wurtz, Fabrice Bauer, Chadi Aludaat, Zoe Demailly, Jean Selim, Vincent Compère, Thomas Clavier, Emmanuel Besnier

**Affiliations:** 1 Department of Anaesthesiology and Critical Care, CHU Rouen, 76031 Rouen, France; 2 Department of Cardiac Surgery, CHU Rouen, 76031 Rouen, France; 3 Rouen Univ, Inserm U1096, EnVi, 76000 Rouen, France; 4 Rouen Univ, Inserm U1239, 76000 Rouen, France; 5 Departement d’Anesthésie-Réanimation, CHU Charles Nicolle, 1 Rue de Germont, 76031 Rouen Cedex, France

**Keywords:** Ventilation, Cardiopulmonary bypass, Lung volume measurement, Functional residual capacity, Cardiac surgery

## Abstract

**Background:**

Many studies explored the impact of ventilation during cardiopulmonary bypass (CPB) period with conflicting results. Functional residual capacity or End Expiratory Lung Volume (EELV) may be disturbed after cardiac surgery but the specific effects of CPB have not been studied. Our objective was to compare the effect of two ventilation strategies during CPB on EELV.

**Methods:**

Observational single center study in a tertiary teaching hospital. Adult patients undergoing on-pump cardiac surgery by sternotomy were included. Maintenance of ventilation during CPB was left to the discretion of the medical team, with division between "ventilated" and "non-ventilated" groups afterwards. Iterative intra and postoperative measurements of EELV were carried out by nitrogen washin-washout technique. Main endpoint was EELV at the end of surgery. Secondary endpoints were EELV one hour after ICU admission, PaO_2_/FiO_2_ ratio, driving pressure, duration of mechanical ventilation and post-operative pulmonary complications.

**Results:**

Forty consecutive patients were included, 20 in each group. EELV was not significantly different between the ventilated versus non-ventilated groups at the end of surgery (1796 ± 586 mL vs. 1844 ± 524 mL, *p* = 1) and one hour after ICU admission (2095 ± 562 vs. 2045 ± 476 mL, *p* = 1). No significant difference between the two groups was observed on PaO_2_/FiO_2_ ratio (end of surgery: 339 ± 149 vs. 304 ± 131, *p* = 0.8; one hour after ICU: 324 ± 115 vs. 329 ± 124, *p* = 1), driving pressure (end of surgery: 7 ± 1 vs. 8 ± 1 cmH_2_O, *p* = 0.3; one hour after ICU: 9 ± 3 vs. 9 ± 3 cmH_2_O), duration of mechanical ventilation (5.5 ± 4.8 vs 8.2 ± 10.0 h, *p* = 0.5), need postoperative respiratory support (2 vs. 1, *p* = 1), occurrence of pneumopathy (2 vs. 0, *p* = 0.5) and radiographic atelectasis (7 vs. 8, *p* = 1).

**Conclusion:**

No significant difference was observed in EELV after cardiac surgery between not ventilated and ventilated patients during CPB.

**Supplementary Information:**

The online version contains supplementary material available at 10.1186/s13019-022-02063-7.

## Introduction

Pulmonary complications are the second most common cause of morbidity after cardiac surgery [1]. They lead to increased length of stay and mortality [2, 3]. After cardiac surgery, alteration of respiratory function is observed during the immediate postoperative period and can last several days [4]. This is characterized by a decrease in lung volumes and especially in functional residual capacity (FRC), which can lead to atelectasis.

A major mechanism for FRC reduction is related to the functional alteration of the diaphragm muscle, secondary to several mechanisms, including supine position, general anesthesia and direct surgical damages [5]. These mechanisms are particularly present during cardiac surgery because of the thoracic surgical approach, the duration of anesthesia and mechanical ventilation, but are not specific.

One of the specificities of cardiac surgery is the use of Cardio-Pulmonary Bypass (CPB). During CPB, the inflation of pulmonary volumes, notably with the use of positive end-expiratory pressure (PEEP), can impede the surgeon, particularly during sternal access. Thus, up to 50% of some anesthetists systematically suspend mechanical ventilation during the CPB period to improve surgical comfort, the oxygenation and gas exchange being ensured by the heart–lung machine [6, 7]. In recent years, numerous studies have been carried out to explore the relevance of maintaining (or not) the mechanical ventilation during CPB, however their results are conflicting and have not led to any guidelines on the subject [8–10]. Indeed, a close relationship has been observed between non-ventilated lung volumes and the degree of hypoxemia, shunt and pulmonary arterial hypertension in non-selected ICU patients [11]. Thus, it appears, at least from an intuitive point of view, that maintaining lung volumes, especially FRC, may be of major interest to prevent the formation of atelectasis and the subsequent pulmonary complications [12, 13].

Thus, we hypothesized that the maintenance of mechanical ventilation during CPB allows a better preservation of FRC. The main objective of our study was to explore the effect of mechanical ventilation during CPB on FRC, evaluated by end-expiratory lung volume. Secondary objectives were the impact of this ventilation strategy on oxygenation and pulmonary outcomes. We also investigated other parameters such as oxygenation, occurrence of pneumonia, atelectasis on chest X-ray, and use of intensive respiratory support.

## Materials and methods

### Study characteristics

We conducted an observational, non-randomized and single center study over a three-month period, from April to July 2020, in the cardiac surgical unit of a tertiary care teaching hospital. Consecutive patients operated by four different senior surgeons were included, under the condition that the equipment for lung volumes measurement was available. This study was approved by our local ethics committee (*Comité d’Ethique pour la Recherche Non-Interventionnelle,* Rouen University Hospital, chairperson Pr Luc-Marie Joly, approval n°E2021-27). Because of the non-interventional design of the study and according to French law [14], no written consent was required and no pre-registration on a public research website was realized. The perioperative strategy of ventilation for the non-CPB period of cardiac surgery was protocolized as a part of our local practices, as described hereafter before data acquisition.

### Population

Patients were eligible if they underwent cardiac surgery by median sternotomy, with CPB and aortic clamping, under general anesthesia, with tracheal intubation and invasive mechanical ventilation using the Carescape R860® ventilator (General Electric Healthcare, USA) required for FRC estimation. Non-inclusion criteria were pregnancy, morbid obesity (BMI > 40 kg/m2), chronic lung or neuromuscular disease, heart transplant procedure, cardiac assist device implantation, aortic dissection or surgery requiring a lateral thoracic approach. Our surgical team was constituted of 4 senior surgeons and 6 senior anesthetists. Among them two used to perform surgery with the continuation of a minimal mechanical ventilation during CPB, and two request the interruption of the ventilation because they estimated that it impede surgical access, especially for sternotomies. Thus, we have included all eligible consecutive patients in these two groups of surgeons and when the GE Carescape R860® ventilator was available (see below). Patients were excluded from analysis in case of a prolonged interruption of ventilation (> 5 min) during CPB, in cases of direct surgical damage to the lungs or evidence for pulmonary aspiration during anesthesia. Ventilation strategy has been previously standardized in our unit to homogenize practices among anesthetists (see section *Intraoperative care and ventilation strategies)*.

### Intraoperative care and ventilation strategies

After admission in operative room, patients were monitored as usual for high-risk general anesthesia: electrocardioscopy, pulse oximetry, invasive blood pressure using a radial 4F catheter and central venous pressure through the jugular central venous catheter. Pre-oxygenation was carried out in a strict supine position (0°) using a 100% FiO_2_ until obtaining an expired fraction of oxygen ≥ 90%. Pre-oxygenation modality was left at the appreciation of the anesthetist between spontaneous ventilation or spontaneous ventilation with inspiratory support (4–10 cmH_2_O to achieve a 7 mL/kg tidal volume, PEEP 4–6 cmH2O). Induction was then carried out by a combination of hypnotics (target-controlled infusion (TCI) of propofol or etomidate 0.4 mg/kg or ketamine 2 mg/kg), opioids (TCI of Sufentanil or Remifentanil) and neuromuscular blocking agents (cisatracurium 0.2 mg/kg). Anesthesia was then maintained by TCI of propofol and of the same opioid that was used for induction. Neuromuscular blockade was maintained by continuous administration of cisatracurium 0.1 mg/kg/h. Depth of anesthesia was assessed by BiSpectral Index Monitoring (BIS, Covidien, France) with a 40–60 target. Ventilation with a face mask was carried out with volume-controlled ventilation mode (Vt 7 mL/kg of ideal body weight, respiratory rate 12–18/min, PEEP 5cmH_2_O and FiO_2_ 100%). Orotracheal intubation was then performed using direct laryngoscopy by a senior anesthetist. After intubation, mechanical ventilation was continued on the GE Carescape R860® ventilator. A standardized alveolar recruitment maneuver was then systematically performed, with controlled pressure ventilation mode and progressive increase in PEEP and insufflation pressures (see Additional file 1), with a maximum level of 20 + 12 cmH_2_O maintained for 10 cycles before a progressive decrease. Ventilation was then carried out in volume-controlled mode (tidal volume 6–8 mL/kg, rate adjusted for ETCO_2_ 28–35 mmHg, FiO_2_ for SpO2 ≥ 95%, PEEP 4–6 cmH_2_O, I:E ratio 1:2). During sternal sawing, according to our habits, ventilation was suspended (< 30 s), without PEEP and without disconnection from the ventilator, to limit the risk of pulmonary lesions. Maintenance of ventilation during CPB was left to the discretion of the medico-surgical team according to habits and surgical difficulties (see above). Patients were retrospectively categorized as "ventilation" and "non-ventilation" groups afterwards, according to the application of ventilation or not during CPB. The per-CPB ventilation consisted in a 2 mL/kg tidal volume, a 8/min respiratory rate, a 5 cmH_2_O PEEP and a 40% FiO_2_ with an inspiratory/expiratory ratio of 1:1. This small tidal volume ventilation was applied during the whole aortic clamping period. In the “non-ventilation group”, patients were still connected to the ventilation machine but no fresh gas flow, no tidal volume and no PEEP were applied during the whole aortic clamping. An alveolar recruitment maneuver was performed immediately after removal of aortic clamp and pre-CPB ventilation was then resumed before weaning from CPB, with an initial FiO_2_ of 80% quickly adjusted to obtain SpO_2_ ≥ 95%.

During the procedure, the GE Carescape R860® ventilator coupled with the dedicated gas analyzer allowed continuous measurements of N_2_ and the subsequent measurement of End-Expiratory Lung Volume (EELV), which approximate FRC in the presence of PEEP, using the nitrogen "wash in-wash out" technique. It consists of a continuous measurement of changes in O_2_ and CO_2_ concentrations when the FiO_2_ changes and until equilibrium (washin), in order to calculate the dilution of N_2_ and deduce the EELV, which approximates FRC in the presence of PEEP. The EELV measurement is the average of this measurement (washin) and a second one, taken when the basic FiO_2_ is returned to baseline (washout) [15]. For each patient, we collected a baseline measurement after intubation and alveolar recruitment maneuver (T0), after chest-closure measurement (T1) and 1 h after ICU admission (T2). Each measurement was performed after a stabilization period of at least 5 min with no change in ventilation parameters as recommended for EELV measurement. An arterial blood gas measure was realized at each time-point as usual (for design representation, see Additional file 2).

The transfer from operative room to intensive care unit (ICU) was performed using Monnal T60® transport ventilator. To avoid de-recruitment events during disconnections, an alveolar recruitment maneuver was performed and then circuit changes were performed after the intubation tube was clamped during an expiratory pause. In the ICU, these patients were ventilated with GE Carescape R860® ventilator, allowing monitoring of EELV, with 6 mL/kg tidal volume, 5–8 mmHg PEEP and respiratory rate adjusted for PaCO_2_ 4–6 kPa. An alveolar recruitment maneuver was performed at the admission in ICU at the exception of patients with circulatory failure. The study protocol is presented in the Additional file 2.

All tidal volumes during this study were adjusted to the ideal body weight (IBW) calculated as IBW = *X* + 0.91 (height in cm − 152.4) with *X* = 50 for men and *X* = 45.5 for women.

### Endpoints

The primary endpoint was the value of EELV at the end of the procedure, after chest closure (T1).

The secondary end points were EELV 1 h after ICU admission (T2), and different parameters of blood gases and pulmonary pressures at T1 and T2: PaO_2_, PaO_2_/FiO_2_, PaCO_2_, Driving Pressures (plateau pressure − PEEP) and static lung compliance (Cstat). We also explored the impact of the two strategies on pulmonary outcomes during ICU stay: use of respiratory support (non-invasive ventilation, high-flow nasal cannula or invasive mechanical ventilation for acute respiratory failure), and occurrence of pneumonia (clinically suspected or confirmed by bacteriological samples). We also explored the presence of atelectasis at day-one after surgery by systematic screening of chest X-ray, evaluated a posteriori by an expert radiologist blinded to the intraoperative strategy. No blinding was ensured for the staff in ICU or operative room.

### Statistical analyses

As this is a pilot study with no data available from the literature, no calculation of the number of subjects was realized. Because of the physiologic endpoint, we estimated that 20 patients per group may be sufficient to demonstrate a difference.

The normality of the two groups was investigated by a D’Agostino test and data are presented as median with first and third quartiles or mean with standard deviations for quantitative variables, or percentage for qualitative variables. Because of the normal distribution of data for EELV, comparisons of quantitative variables over time were carried out using a two-way ANOVA test for matched values considering time (T0, T1 and T2) and group (ventilated or non-ventilated). Holm-Sidak post-test for multiple comparisons was performed between the ventilated and non-ventilated groups at each time-point to adjust *p* value to the number of comparisons. Comparisons of descriptive quantitative variables between the two groups were performed using a Student t-test or a Mann and Whitney two-tailed test. Comparisons of qualitative variables were performed using an exact Fischer's test. An adjusted *p* value strictly inferior to 0.05 was considered significant. Analyses were performed using GraphPad Prism v8.2 (GraphPad, USA).

## Results

From April 10th to July 17th 2020, we included a total of 40 patients (20/group). Demographic characteristics of the population are summarized in Table 1 with no difference concerning the various data. The use of positive pressure ventilation as a preoxygenation technique was similar in both groups (*n* = 13 vs. 12, *p* = 1). There was no difference for duration of surgery (196 ± 49 vs. 196 ± 64 min, *p* = 0.9), CPB (86 ± 41 vs. 81 ± 34 min, *p* = 0.7) or aortic clamping (62 ± 30 vs. 61 ± 28 min, *p* = 0.9). No difference was observed concerning the incidence of visualized pleural effraction during surgery (13 vs 14, *p* = 1). Fluids infusion during the operative period was also similar (1700 ± 340 vs 1675 ± 495 mL, *p* = 0.8). Intraoperative ventilator settings in both groups are presented in Additional file 3. Per-CPB ventilator settings in the “ventilation” group were 2.6 ± 0.6 mL/kg, 9 ± 2 cycles/min, 5 ± 1 cmH_2_O PEEP and 40 ± 10% FiO_2_ and no interruption in the ventilation was observed.

**Table 1:** preoperative demographic characteristics of the population

	Ventilation	No ventilation	*p* value
Age (years)	70 [66–74]	71 [64–75]	0.9
Male gender (*n*, %)	11 (55%)	16 (80%)	0.2
BMI (kg/m^2^)	25.9 ± 3.9	26.7 ± 4.2	0.5
BMI ≥ 30 kg/m^2^	6 (30%)	7 (35%)	1
Hypertension	14 (70%)	16 (80%)	0.7
Stroke	1	3	–
Smoking status	9 (45%)	8 (40%)	1
COPD	1	2	–
Obstructive sleep apnea	1	6	–
Preoperative Kidney disease	4	6	0.7
Diabetes	4	8	0.3
Euroscore II (%)	1.6 [1.0–2.3]	1.7 [1.1–2.9]	0.8
NYHA	2 ± 1	2 ± 1	0.8
Preoperative LVEF (%)	65 [51–71]	66 [63–70]	0.3
Type of surgery:			0.08
Coronary artery bypass	11	6	
Valvular surgery	8	8	
Combined procedures	1	6	
Theoretical FRC (mL)	3472 ± 207	3587 ± 173	0.06
Preoperative SpO_2_ (%)	95 ± 7	97 ± 2	0.1

*BMI*: Body Mass Index, *COPD*: Chronic Obstructive Pulmonary Disease, *FRC*: theoretical functional respiratory capacity estimated by the formula FRC (L) = 2.34 × height (m) + age × 0.01–1.09 (20), *LVEF*: Left Ventricular Ejection Fraction

Results for the primary and secondary endpoints are presented in Table 2. Briefly, there was no difference concerning EELV at the end of surgery (1796 ± 566 vs 1844 ± 524 mL, *p* = 0.6) but also at the other time-points, *i.e.*at baseline (after orotracheal intubation) and 1 h after ICU admission (2095 ± 562 vs 2045 ± 476 mL, *p* = 1). There was also no difference concerning static compliance (Cstat), driving pressure, blood oxygenation (PaO_2_/FiO_2)_ and CO_2_ partial pressure at the three time-points. The main outcomes in ICU were not different between “ventilation” and “no-ventilation” groups for simplified acute physiology score 2 (40 ± 15 vs. 36 ± 8, *p* = 0.3), sepsis-related organ failure assessment at admission (4 ± 2 vs. 4 ± 1), duration of postoperative mechanical ventilation (5.5 ± 4.8 vs. 8.2 ± 10.0 h, *p* = 0.5), incidence of X-ray atelectasis (7 vs. 8, *p* = 1), use of high-flow nasal cannula or non-invasive ventilation support (2 vs. 1, *p* = 1) or pneumonia (2 vs. 0, *p* = 0.5) (Table 3).

**Table 2:** Results for physiologic parameters in “ventilation” and “no-ventilation” CPB strategies at the three time-points T0 (baseline after orotracheal intubation), T1 (end of surgery) and T2 (1 h after ICU admission). EELV: End Expiratory Lung Volumes; Cstat: static compliance

	T0			T1			T2		
	Ventilation	No-ventilation	*p* value	Ventilation	No-ventilation	*p* value	Ventilation	No-ventilation	*p* value
EELV (mL)	2206 ± 790	1966 ± 482	0.6	1796 ± 566	1844 ± 524	1	2095 ± 562	2045 ± 476	1
Cstat (mL/cmH_2_O)	73 ± 35	79 ± 25	0.9	71 ± 27	66 ± 16	0.9	57 ± 15	58 ± 14	1
Driving Pressure (cmH_2_O)	8 ± 3	7 ± 2	0.6	7 ± 1	8 ± 1	0.3	9 ± 3	9 ± 3	1
PaO2/FiO2 (mmHg)	381 ± 156	398 ± 137	1	339 ± 149	304 ± 131	0.8	324 ± 115	329 ± 124	1
PaCO2 (mmHg)	40 ± 4	38 ± 5	0.6	37 ± 4	37 ± 4	1	39 ± 4	39 ± 4	1

**Table 3:** Postoperative clinical outcomes between groups

	Ventilation	No ventilation	*p* value
SAPS-2 at admission	40 ± 15	36 ± 8	0.3
SOFA at admission	4 ± 2	4 ± 1	1
Duration of mechanical ventilation (in hours)	5.5 ± 4.8	8.2 ± 10.0	0.5
Use of NIV or HFNC (*n*)	2	1	1
Pneumonia (*n*)	2	0	0.5

HFNC: high-flow nasal cannula; NIV: non-invasive ventilation; SAPS-2: severity acute physiology score 2; SOFA: sepsis-related organ failure assessment

## Discussion

Maintaining mechanical ventilation during CPB was not associated in our study with better preservation of EELV. Despite less accurate than chest tomography imaging, N_2_ washin-washout techniques have been widely studied and showed good correlations and/or agreements with pulmonary volumes, allowing their use at the bench side in various settings, including critical cares [16–18]. We can therefore strongly believe that post-operative FRC was not modified by per-CPB ventilation in our cohort.

This study approaches the effect of per-CPB ventilation from an original angle. Previous studies have focused on its effect on oxygenation, postoperative respiratory complications or systemic inflammation [8, 10, 19]. To date there is no study specifically studying the impact of maintaining mechanical ventilation per-CPB on lung volumes during adult cardiac surgery, whereas most of the modern ventilation strategies in ICU or operative room are based on this concept of “lung protection”, using reduced tidal volume, sufficient PEEP and recruitment maneuvers to prevent lung collapse [20, 21].

Different studies have investigated the effect of maintaining ventilation during CPB on different clinical or biological parameters. A meta-analysis of 15 randomized trials conducted between 1993 and 2016, explored mechanical ventilation or continuous positive airway pressure during CPB on the evolution of the alveolar-arterial oxygen gradient, oxygenation, duration of mechanical ventilation and length of stay [22]. No significant difference was found with regard to the criteria studied. However, a significant improvement of the PaO_2_/FiO_2_ ratio in favor of maintaining ventilation can be noted in one of the 4 trials studying this parameter, as well as a decrease of the alveolo-arterial oxygen gradient in 2 trials. One of the limits of this meta-analysis was the heterogeneity of ventilation parameters among studies. More recently, the PROVECS study compared the effect of two ventilation strategies on the occurrence of postoperative respiratory complications. A total of 494 patients was randomized between "conventional" ventilation strategy and "protective" strategy, which included the maintenance of ventilation during CPB but also higher intraoperative PEEP and recruitment maneuvers. No significant difference was found in the occurrence of postoperative pulmonary complications, hypoxemia or use of respiratory support. In this trial, control group, with 2 cmH_2_O PEEP without ventilation during CPB, was compared with an “open-lung” group, with 8 cmH_2_O PEEP, 12 respiratory rate and FiO_2_ 40%. Two elements may have been discussed and may underlie the absence of beneficial effects. First, 2 cmH_2_O PEEP is not “no PEEP” and may already have little effects on FRC. Secondly, population in this trial presented few risks of pulmonary complications. Thus, the effects may have been different in patients at risk of postoperative pulmonary complications, with elevated preoperative risk scores such as Gupta or ARISCAT. For the same reasons, we cannot rule out that different results may have been observed in our study with a more selected “at risk” population. Contrariwise, a recent randomized study showed that the use of ventilation with low FiO_2_ (30%) during CPB was associated with less incidence of severe post-operative pulmonary complications than no ventilation (29 vs. 59%) [23]. Differences between studies (including ours) may explain this discrepancy. First, patients included in this latter study presented higher pulmonary risks, with a significant proportion of active smokers, in combination with longer duration of CPB, which may potentiate the risk for atelectasis. Secondly, only one alveolar recruitment maneuver was scheduled in this trial, whereas we applied these maneuvers at least four times in our protocol. Thus, we can hypothesize that alveolar recruitment is more effective in preventing atelectasis than a continuous ventilation. This trial confirmed that application of a low FiO_2_ seems more beneficial, probably by preventing atelectasis formation due to O_2_ resorption.

Different physiological hypotheses may explain the lack of effect in our study. The duration of CPB was limited with a mean duration of 60 min, and thus we can hypothesize that this period was too short to induce a significant difference in FRC. Another element may be the non-selected nature of the included patients, notably with the absence of patients with pulmonary underlying conditions. Indeed, in the IMPROVE study which demonstrated a benefit of protective ventilation in major abdominal surgery, the included patients presented a preoperative risk index for pulmonary complications of more than two [20]. Thus, it is possible that the association of a short duration of aortic clamping and the absence of preoperative risk factors may have mitigated the potential beneficial effects of per-CPB ventilation strategy. Another hypothesis concerns the choice of mechanical ventilation parameters during CPB. In our study, the ventilation was maintained with low tidal volume (Vt 2.6 ± 0.6 mL/kg of IBW), which may be close to the theoretical dead-space volume (≈1.5 mL/kg), and thus insufficient to keep the alveoli opened. However, higher volumes hamper the surgeon, potentially decreasing the quality of surgical exposure. Another element, specific to cardiac surgery under CPB with aortic clamping, is the interruption of pulmonary perfusion. Indeed, experimental studies suggested that pulmonary vascularization is involved in maintaining the mechanical and architectural properties of the lung. Gibney et al., observed in animal models a decrease in pulmonary compliance if vascularization is stopped [24]. Thus, the mere maintenance of mechanical ventilation may not be able to counteract this “ischemic” closure of the lung.

Concerning the secondary endpoints, maintaining mechanical ventilation during CPB does not appear to be associated with any significant effect in our population. Thus, we found no significant difference either on clinical criteria (occurrence of pneumonia, duration of invasive mechanical ventilation, non-invasive ventilation or high flow oxygen therapy) or other criteria (oxygenation parameters, driving pressure, static compliance, postoperative atelectasis). Nevertheless, the small size of the population does not allow us to definitively conclude on this point.

Some limitations of our study must be discussed. First, the lack of randomization limits the impact of conclusion and the generalization of data, despite great similarities between groups, notably concerning euroscore-2, obesity, age, etc*.* Even if we observed a trend to an unbalance concerning the type of surgery, with more combined procedures in the not-ventilated group, this difference was not significant. Because of the limited sample size of this population, we cannot rule out that it induced a selection bias during analysis. Furthermore, if we suggest that the complexity of the procedure may alter pulmonary function, thus this difference may be more susceptible to worsen the EELV in the not-ventilated group, which has not been observed. Moreover, because the choice to maintain or not ventilation during CPB was left to the discretion of the medical team, it may be a source of selection bias. Another point may be the use of N_2_ washin-washout method to explore EELV. Nevertheless, this technique seems to present good accuracy (< 100 mL difference) and reproducibility (< 4% of variations), with a strong correlation with method of reference, even in patients with acute respiratory distress syndrome [17]. Then, we did not study the long-term impact of our strategies, which may have been different between the two strategies. Finally, we cannot rule out a lack of power related to the limited sample size. We did not calculate an a priori number of patients to include because of the absence of previous published data. Nevertheless, based on a study from Dyhr et al.exploring different strategies of ventilation in hypoxemic patients after cardiac surgery, where EELV at baseline was 1080 ± 325 mL, we a posteriori calculated that the inclusion of 32 patients would have allow to observe a 30% difference in EELV with a power of 80% and an alpha risk of 5% [25]. Thus, we estimate that a major difference may have been observed in our study with the current number of patients included.

## Conclusion

The effects of maintaining mechanical ventilation during cardiopulmonary bypass are still controversial to this day. In non-randomized study, this strategy with a tidal volume of 2 mL/kg with PEEP does not seem to allow a better preservation of EELV after cardiac surgery.

## Supplementary Information

**Additional file 1.** Modalities of alveolar recruitment maneuvres.

**Additional file 2.** Study design.

**Additional file 3.** Respiratory settings in the two groups.

## Data Availability

The datasets generated and/or analysed during the current study are not publicly available but are available from the corresponding author on reasonable request.

## References

[1] Szelkowski LA, Puri NK, Singh R (2015). Current trends in preoperative, intraoperative, and postoperative care of the adult cardiac surgery patient. Curr Probl Surg.

[2] Weissman C (2004). Pulmonary complications after cardiac surgery. Semin Cardiothorac Vasc Anesth.

[3] Welsby IJ, Bennett-Guerrero E, Atwell D (2002). The association of complication type with mortality and prolonged stay after cardiac surgery with cardiopulmonary bypass. Anesth Analg.

[4] Lindberg P, Gunnarsson L, Tokics L (1992). Atelectasis and lung function in the postoperative period. Acta Anaesthesiol Scand.

[5] Brismar B, Hedenstierna G, Lundquist H (1985). Pulmonary densities during anesthesia with muscular relaxation—a proposal of atelectasis. Anesthesiology.

[6] Fischer M-O, Courteille B, Guinot P-G (2016). Perioperative ventilatory management in cardiac surgery: a French nationwide survey. Med (Baltimore).

[7] Passaroni AC, de Silva MAM, Yoshida WB (2015). Cardiopulmonary bypass: development of John Gibbon’s heart-lung machine. Rev Bras Cir Cardiovasc.

[8] Loeckinger A, Kleinsasser A, Lindner KH (2000). Continuous positive airway pressure at 10 cm H_2_O during cardiopulmonary bypass improves postoperative gas exchange. Anesth Analg.

[9] Bignami E, Guarnieri M, Saglietti F (2016). Mechanical ventilation during cardiopulmonary bypass. J Cardiothorac Vasc Anesth.

[10] Lagier D, Fischer F, The PROVECS Study Group (2019). Effect of open-lung vs conventional perioperative ventilation strategies on postoperative pulmonary complications after on-pump cardiac surgery: the PROVECS randomized clinical trial. Intensive Care Med.

[11] Gattinoni L, Pesenti A (2005). The concept of “baby lung”. Intensive Care Med.

[12] Layon J, Banner MJ, Jaeger MJ (1986). Continuous positive airway pressure and expiratory positive airway pressure increase functional residual capacity equivalently. Chest.

[13] Rylander C, Högman M, Perchiazzi G (2004). Functional residual capacity and respiratory mechanics as indicators of aeration and collapse in experimental lung injury. Anesth Analg.

[14] Toulouse E, Lafont B, Granier S (2020). French legal approach to patient consent in clinical research. Anaesth Crit Care Pain Med.

[15] Olegård C, Söndergaard S, Houltz E (2005). Estimation of functional residual capacity at the bedside using standard monitoring equipment: a modified nitrogen washout/washin technique requiring a small change of the inspired oxygen fraction. Anesth Analg.

[16] Chiumello D, Cressoni M, Chierichetti M (2008). Nitrogen washout/washin, helium dilution and computed tomography in the assessment of end expiratory lung volume. Crit Care Lond Engl.

[17] Dellamonica J, Lerolle N, Sargentini C (2011). Accuracy and precision of end-expiratory lung-volume measurements by automated nitrogen washout/washin technique in patients with acute respiratory distress syndrome. Crit Care Lond Engl.

[18] Berger-Estilita J, Haenggi M, Ott D (2021). Accuracy of the end-expiratory lung volume measured by the modified nitrogen washout/washin technique: a bench study. J Transl Med.

[19] Ng CSH, Arifi AA, Wan S (2008). Ventilation during cardiopulmonary bypass: impact on cytokine response and cardiopulmonary function. Ann Thorac Surg.

[20] Futier E, Constantin J-M, Paugam-Burtz C (2013). A trial of intraoperative low-tidal-volume ventilation in abdominal surgery. N Engl J Med.

[21] Mercat A, Richard J-CM, Vielle B (2008). Positive end-expiratory pressure setting in adults with acute lung injury and acute respiratory distress syndrome: a randomized controlled trial. JAMA.

[22] Wang Y-C, Huang C-H, Tu Y-K (2018). Effects of positive airway pressure and mechanical ventilation of the lungs during cardiopulmonary bypass on pulmonary adverse events after cardiac s urgery: a systematic review and meta-analysis. J Cardiothorac Vasc Anesth.

[23] Zhang MQ, Liao YQ, Yu H (2021). Effect of ventilation strategy during cardiopulmonary bypass on postoperative complications after cardiac surgery: a randomized clinical trial. J Cardiothorac Surg.

[24] Gibney BC, Wagner WL, Ysasi AB (2017). Structural and functional evidence for the scaffolding effect of alveolar blood vessels. Exp Lung Res.

[25] Dyhr T, Nygard E, Laursen N, Larsson A (2004). Both lung recruitment maneuver and PEEP are needed to increase oxygenation and lung volume after cardiac surgery. Act Anaesthesiol Scand.

