# Supplementary figures and images for: Effect of mechanical ventilation during cardiopulmonary bypass on end-expiratory lung volume in the perioperative period of cardiac surgery: an observational study
Source: J Cardiothorac Surg. 2022 Dec 22;17:331. doi: 10.1186/s13019-022-02063-7 (PMC9784092; doi:10.1186/s13019-022-02063-7)

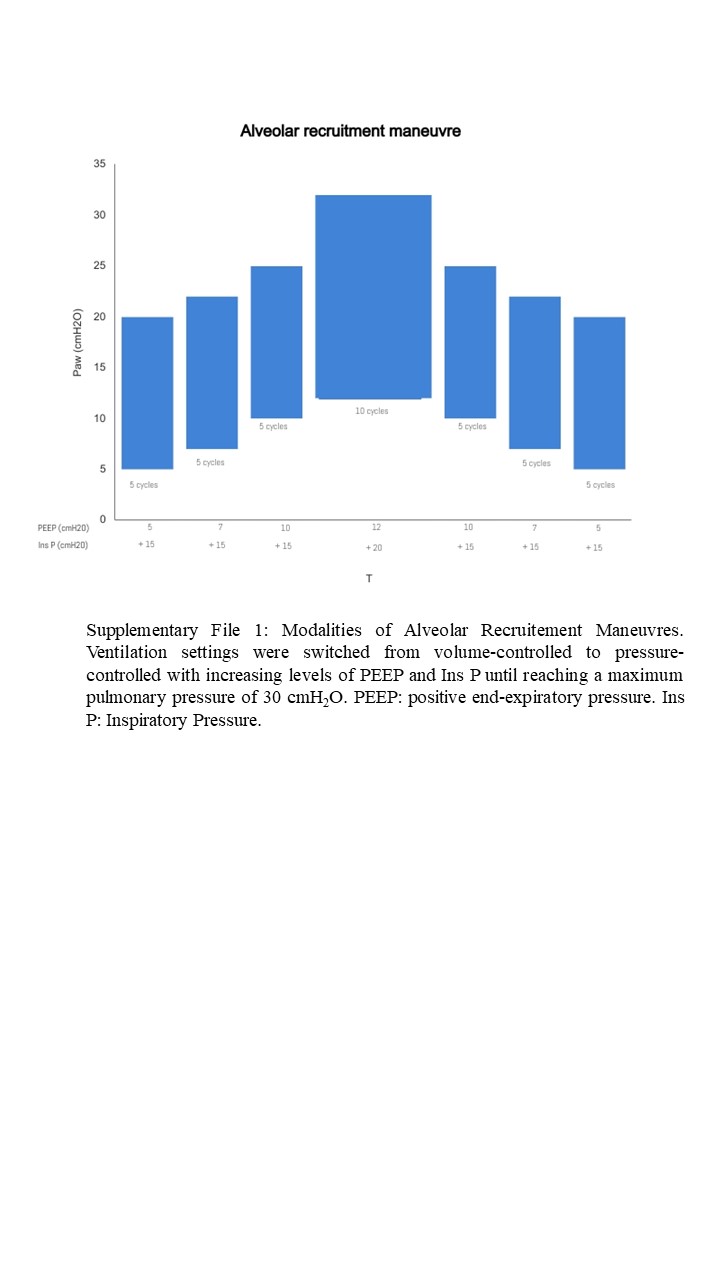

Supplement: Supplementary file 1 — Additional file 1. Modalities of alveolar recruitment maneuvres. [file 13019_2022_2063_MOESM1_ESM.jpg]

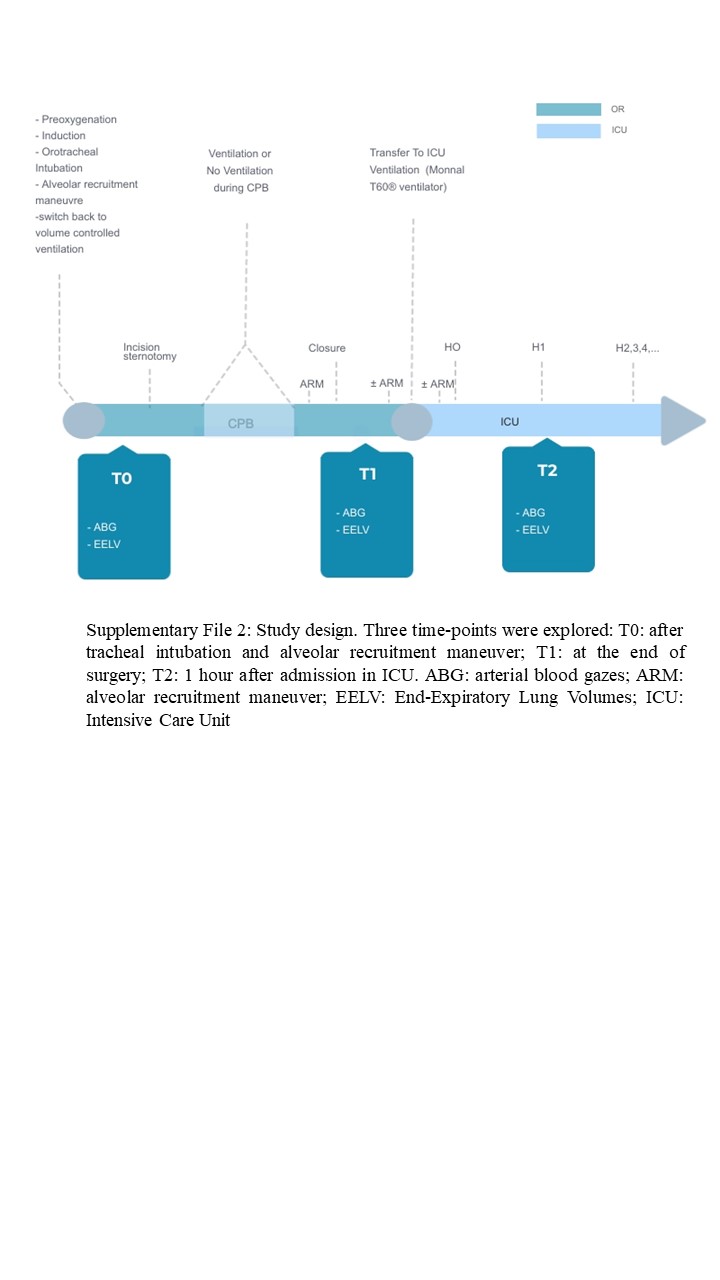

Supplement: Supplementary file 2 — Additional file 2. Study design. [file 13019_2022_2063_MOESM2_ESM.jpg]
