# Supplementary material for: Effect of mechanical ventilation during cardiopulmonary bypass on end-expiratory lung volume in the perioperative period of cardiac surgery: an observational study
Source: J Cardiothorac Surg. 2022 Dec 22;17:331. doi: 10.1186/s13019-022-02063-7 (PMC9784092; doi:10.1186/s13019-022-02063-7)
Supplement: Supplementary file 3 — Additional file 3. Respiratory settings in the two groups. [file 13019_2022_2063_MOESM3_ESM.docx]

|  | Ventilation during CPB  (n=20) | No Ventilation during CPB  (n=20) |
| --- | --- | --- |
| Theoretical FRC (mL) | 3472 ± 207 | 3587 ± 173 |
| Preoperative SpO2 (%) | 95 ± 7 | 97 ± 2 |
| Ventilator settings :  T0:  tidal Volume (ml/kg)  RR (/min)  PEEP (cmH2O)  FiO2 (%)  T1:  Tidal volume (ml/kg)  RR (/min)  PEEP (cmH2O)  FIO2 (%)  T2:  Tidal volume (ml/kg)  RR (/min)  PEEP (cmH20)  FiO2 (%) | 7,1 ± 0,8  15 ± 2  6 ± 1,7  63 ± 15  7,1 ± 0,8  16 ± 3  6,3 ± 2,7  58 ± 11  7,2 ± 0,9  17 ± 3  6,1 ± 1,3  44 ± 9 | 7 ± 0,6  15 ± 2  6,5 ± 1,9  66 ± 14  6,9 ± 0,5  15 ± 2  6,4 ± 1,8  63 ± 13  6,9 ± 0,6  17 ± 2  6,4 ± 1,5  45 ± 10 |
| Ventilator settings during CPB:  Tidal volume (mL/kg)  RR (/min)  PEEP (cmH2O)  FiO2 (%) | 2.6 ± 0.6  9 ± 2  5 ± 1  40 ± 10 | -  -  - |

Supplementary File 3: respiratory settings in the 2 groups at the 3 different time-points and during CPB period. PEEP: positive end expiratory pressure; RR: respiratory rate; FRC: functional residual capacity; T0: after orotracheal intubation; T1: end of surgery; T2: 1 hr after ICU admission.
